# Disruption of Immune Homeostasis in Human Dendritic Cells via Regulation of Autophagy and Apoptosis by *Porphyromonas gingivalis*

**DOI:** 10.3389/fimmu.2019.02286

**Published:** 2019-09-24

**Authors:** Mohamed M. Meghil, Omnia K. Tawfik, Mahmoud Elashiry, Mythilypriya Rajendran, Roger M. Arce, David J. Fulton, Patricia V. Schoenlein, Christopher W. Cutler

**Affiliations:** ^1^Department of Periodontics, Dental College of Georgia at Augusta University, Augusta, GA, United States; ^2^Department of Oral Biology and Diagnostic Sciences, Dental College of Georgia at Augusta University, Augusta, GA, United States; ^3^Vascular Biology Center, Medical College of Georgia at Augusta University, Augusta, GA, United States; ^4^Department of Cellular Biology and Anatomy, Medical College of Georgia at Augusta University, Augusta, GA, United States

**Keywords:** (MeSH) dendritic cells, *Porphyromonas gingivalis*, autophagy, apoptosis, periodontitis

## Abstract

As fundamental processes of immune homeostasis, autophagy, and apoptosis must be maintained to mitigate risk of chronic inflammation and autoimmune diseases. Periodontitis is a chronic inflammatory disease characterized by oral microbial dysbiosis, and dysregulation of dendritic cell (DC) and T cell responses. The aim of this study was to elucidate the underlying mechanisms by which the oral microbe *Porphyromonas gingivalis* (*P. gingivalis*) manipulates dendritic cell signaling to perturb both autophagy and apoptosis. Using a combination of Western blotting, flow cytometry, qRT-PCR and immunofluorescence analysis, we show a pivotal role for the minor (Mfa1) fimbriae of *P. gingivalis* in nuclear/cytoplasmic shuttling of Akt and FOXO1 in human monocyte-derived DCs. Mfa1-induced Akt nuclear localization and activation ultimately induced mTOR. Activation of the Akt/mTOR axis downregulated intracellular LC3II, also known as Atg8, required for autophagosome formation and maturation. Use of allosteric panAkt inhibitor MK2206 and mTOR inhibitor rapamycin confirmed the role of Akt/mTOR signaling in autophagy inhibition by *P. gingivalis* in DCs. Interestingly, this pathway was also linked to induction of the anti-apoptotic protein Bcl2, decreased caspase-3 cleavage and decreased expression of pro-apoptotic proteins Bax and Bim, thus promoting longevity of host DCs. Addition of ABT-199 peptide to disrupt the interaction of antiapoptotic Bcl2 and its proapoptotic partners BAK/BAX restored apoptotic death to *P. gingivalis-*infected DC cells. In summary, we have identified the underlying mechanism by which *P. gingivalis* promotes its own survival and that of its host DCs.

## Introduction

Periodontitis is an inflammatory oral disease that affects nearly half of the population in the United States (1). It is characterized by dysbiosis within the oral microbiota (2, 3), which is associated with deregulation of the host immune response. Dysbiotic microbiota prolong the host response, leading to destruction of soft and hard tissues supporting the tooth (4, 5). Dendritic cells (DCs) are antigen-presenting cells (APCs) that circulate throughout the blood and enter peripheral tissues, where they capture microbes via pattern recognition receptors (PRRs) (6, 7). *Porphyromonas gingivalis* (*P. gingivalis*) is recognized as a keystone pathogen through its ability to manipulate the oral microbiome (8). DCs respond locally to *P. gingivalis-*induced dysbiosis (6, 9–11) and carry *P. gingivalis* in the bloodstream to distant sites (12). Several studies have reported the relationship between *P. gingivalis* and systemic diseases, including cardiovascular disease, respiratory tract infections, Alzheimer's disease, diabetes mellitus, and preterm low birth weight babies (13–18). *P. gingivalis* expresses many virulence factors, contributing to its defense and destruction against host tissues and cells, that are sensed by DCs via specific PRRs. Most notable are the adhesion proteins, known as fimbriae. Generally, two types of fimbriae are recognized on the surface of *P. gingivalis*, the major (FimA) fimbria, encoded by *fimA* gene, and minor (Mfa1) fimbria, encoded by *mfa1* gene (19). It has been reported that the *P. gingivalis* minor fimbria target the C-type lectin receptor DC-SIGN (20, 21), while the major fimbria target the Toll-like receptor 2 (TLR2) (22, 23).

Autophagy is central to the development of an efficient balanced immune response. The cellular autophagic machinery breaks down damaged proteins and organelles by sequestering and directing cargo to the lysosome. This same machinery is required for APCs to fight invading pathogens and shape cellular immunity (24) through antigen processing, upregulation of costimulatory molecules and cytokines (25–27). Apoptosis is another essential component of immune homeostasis. Inappropriate execution of apoptosis in immune cells can lead to dire consequences such as development of autoimmune diseases (28). We have recently shown that *P. gingivalis* subverts autophagy to survive in DCs through a mechanism involving the targeting of DC-SIGN by Mfa1 fimbria (29). This is involved in inhibition of apoptosis in host DCs (30) through a mechanism that is not clear.

High throughput RNA sequencing of MoDCs infected with *P. gingivalis* and its isogenic fimbriae deficient mutants, have identified a particular set of genes involved in the regulation of autophagy and apoptosis that are dependent on fimbriae-expression pattern (31). The present work has identified at the protein and functional level, the signaling pathway activated by *P. gingivalis* via its Mfa1 fimbriae, involved in both survival and disruption of immune homeostasis through regulation of both apoptosis and/or autophagy in DCs. We now show a pivotal role for nuclear/cytoplasmic shuttling of Akt/FOXO1 in autophagy downregulation by Mfa1 expressing *P. gingivalis*. Autophagy downregulation occurs concomitantly with an upregulation of Bcl2, decreased caspase-3 activation, and the downregulation of the pro-apoptotic proteins Bax and Bim.

## Materials and Methods

### Ethical Aspects

*In vitro* monocyte-derived DCs (MoDCs) studies were determined by the Human Assurance Committee at Augusta University to be human subject exempt, due to the use of anonymized peripheral blood samples for monocytes.

### Reagents

Primary antibodies against Akt, Phosphorylated Akt (p-Akt) Ser473, FOXO1, p-FOXO1 Thr24, mTOR, p-mTOR Ser2448, Raptor, p-Raptor Ser792, ULK-1, p-ULK-1 Ser757, LC3B, Bax, Bim, Bcl2, Caspase 3, Cleaved caspase 3, Cleaved PARP, β-actin, GAPDH, horseradish peroxidase (HRP)-conjugated anti-mouse, and anti-rabbit secondary antibodies and protease/phosphatase inhibitor cocktail were purchased from (Cell Signaling Technology, Danvers, MA). MK2206 and ABT-199 were purchased from (Selleckchem, Houston, TX). HDAC1 antibody, chloroquine, rapamycin, Pam3csk, erythromycin, tetracycline, RIPA buffer and PVDF membranes were purchased from (Sigma-Aldrich, St. Louis, MO). 4–15% Mini-PROTEAN TGX Precast Protein Gel was from (Bio-Rad Laboratories, Inc., Hercules, CA). RPMI 1640 medium, ProLong™ Gold Antifade Mountant with DAPI, Annexin V FITC Apoptosis Detection Kit and NE-PER™ Nuclear and Cytoplasmic Extraction Reagents were purchased from (Thermo Fisher Scientific, Carlsbad, CA). EasySep™ Human Monocyte Enrichment Kit was from (STEMCELL Technologies Inc., Vancouver, Canada). GM-CSF, IL-4, FBS and antibiotic/antimycotic were from (Gemini Bio Products, West Sacramento, CA). Wilkins-Chalgren anaerobe broth from (Neogen Europe, Ltd., Scotland, UK). Western Lightning ECL Pro Chemiluminescent reagent (PerkinElmer Inc., Waltham, MA). RNeasy kit for RNA isolation was from (Qiagen, Germantown, MD). TaqMan® gene expression primers obtained from Thermo Fisher Scientific were used for Real-Time Quantitative Reverse Transcription PCR (qRT-PCR): *Bcl2* (Assay ID: HS00608023_m1), *Bax* (Assay ID: HS00180269_m1), *Bim* (Assay ID: HS00708019_s1) and *GAPDH* (Assay ID: Hs02758991_g1).

### DCs Generation and Culture

Blood samples were obtained from healthy donors at Shepeard Community Blood Center (Augusta, GA). Monocyte-derived DCs were generated from monocytes isolated from fractions of peripheral blood by negative selection using EasySep™ Human Monocyte Enrichment Kit. After isolation, monocytes were seeded in the presence of GM-CSF (1,000 U/ml) and IL-4 (100 U/ml) at a concentration of 1–2 × 10^5^ cells/ml in RPMI 1640 containing 10% heat inactivated FBS and antibiotic/antimycotic for 5–7 days.

### Culture of *P. gingivalis* Strains and DCs Infection

Three *P. gingivalis* strains were used in this study; Wild-type *P. gingivalis* (Pg381) which expresses both FimA and Mfa1 fimbriae, isogenic Mfa1 fimbria-deficient mutant (MFI) which expresses only FimA fimbriae, and isogenic FimA fimbria-deficient mutant (DPG3) which expresses only Mfa1 fimbriae. *P. gingivalis* strains were maintained anaerobically in (10% H_2_, 10% CO_2_, and 80% N_2_) in a Coy lab vinyl anaerobic chamber (Coy Laboratory Products, Inc., Grass Lake, MI) at 37°C in Wilkins-Chalgren anaerobe broth. Mutant strains were maintained using erythromycin (5 μg/ml) for mutant DPG3 and tetracycline (2 μg/ml) for mutant MFI (32). Bacteria suspension was washed five times and resuspended in PBS. CFUs were standardized at an OD of 0.11 at 660 nm (equivalent to 5 × 10^7^ CFU/ml) by spectrophotometry (33). DCs were pulsed with all *P. gingivalis* strains for either 6 or 12 h at a multiplicity of infection (MOI) = 1.

### Total, Nuclear and Cytoplasmic Lysate Extraction

Total cell lysates were extracted by addition of RIPA buffer supplemented by protease/phosphatase inhibitor cocktail and incubation for 20 min on ice. Samples were centrifuged and the supernatant was collected. The nuclear and cytoplasmic fractionation was done by using NE-PER™ Nuclear and Cytoplasmic Extraction Reagents following the manufacturer's protocol.

### Western Blot

Cellular protein was loaded into 4–15% Mini-PROTEAN TGX Precast Protein Gel and transferred to PVDF membranes. The membranes were blocked with 5% milk in TBST, followed by incubation overnight at 4°C with 1:1,000 dilution of primary antibodies. After membranes were washed three times in TBST, horseradish peroxidase-conjugated secondary antibodies were added at a 1:2,000 dilution and incubated for 1 h at room temperature. After three more washes with TBST, the immunoreactive peptide was detected by Western Lightning ECL Pro Chemiluminescent reagent and imaged using ChemiDoc™ MP Imaging System (Bio-Rad Laboratories, Inc., Hercules, CA).

### Flow Cytometric Analysis

Annexin V and propidium iodide (PI) detection was conducted by using eBioscience™ Annexin V FITC Apoptosis Detection Kit, according to the manufacturer's recommendation. Briefly, cells were harvested and washed once with PBS and once with 1X Annexin V binding buffer. Cells were resuspended with 1X binding buffer followed by incubation with Annexin V for 15 min at room temperature in the dark. After washes, samples were resuspended in 1X binding buffer and PI was added before running the samples by using MACSQuant® Analyzer 10 flow cytometer (Miltenyi Biotec Inc., Auburn, CA).

### Immunofluorescence

Cell suspensions were deposited onto glass slides and centrifuged using Cytospin™ 4 Cytocentrifuge (Thermo Fisher Scientific, Carlsbad, CA). Cells were fixed with 4% paraformaldehyde for 10 min, washed with PBS, permeabilized with 0.1% Triton X-100 in PBS for 10 min and blocked for 30 min with 2% BSA in PBS. Primary antibody LC3 from was added to the cells and incubated overnight at 4°C. After washes, secondary antibody was added for 1 h, washed with PBS and slides were mounted with ProLong™ Gold Antifade Mountant with DAPI.

### RNA Isolation and qRT-PCR

Total RNA was extracted from DCs using RNeasy kit following the manufacturer's protocol. Total RNA was reverse transcribed into cDNA. The cDNA was then amplified by PCR using the High-Capacity cDNA Reverse Transcription Kit® with random primers in total reaction of 20 μL.

### Statistical Analysis

Data analysis was conducted by one-way ANOVA followed by Tukey's multiple-comparisons test using GraphPad Prism 6 (GraphPad Software, Inc, La Jolla, CA). Values are expressed as mean ± standard deviation (SD). Experiments were carried out a minimum of three times.

## Results

### Differential Nuclear and Cytoplasmic Shuttling of Akt and FOXO1 and Attenuation of TLR1/2 Signal in *P. gingivalis*-Infected DCs

**Figure 1:**
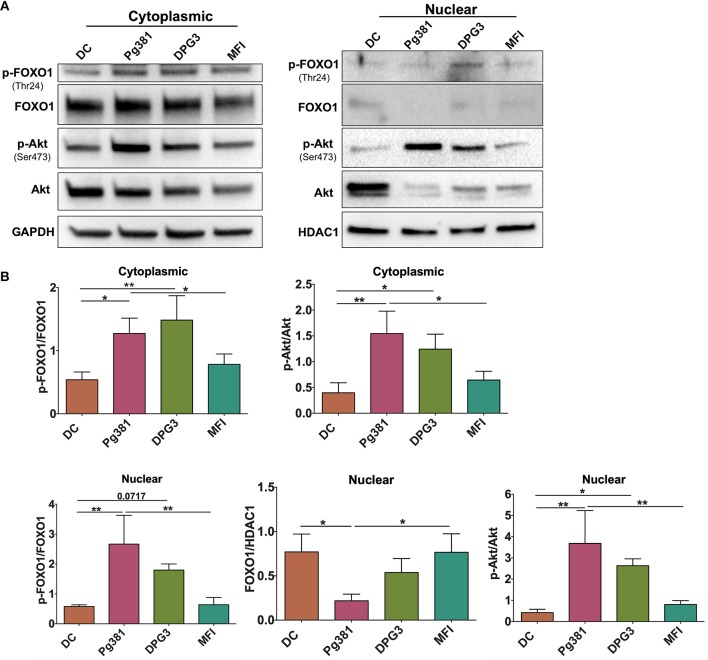
*P. gingivalis* minor fimbria induces phosphorylation of Akt and FOXO1. DCs were infected with *P. gingivalis* strains, Pg381, DPG, and MFI for 6 h. Cytoplasmic and nuclear fractions were isolated from all groups. **(A)** Representative images of immunoblot analysis of Akt, p-Akt, FOXO1, and p-FOXO1 using cytoplasmic and nuclear extracts from uninfected DCs and DC infected with *P. gingivalis* strains. **(B)** Densitometric analysis of Akt, p-Akt, FOXO1, and p-FOXO1 in the cytoplasm and nucleus. After normalization of the phosphorylated protein with the corresponding total protein, the protein signal of control was set as 1, and the signals of other groups were normalized with control to calculate fold changes. GAPDH and HDAC1 were used as internal control for normalizing the data. Data are expressed as mean ± SD. *P < 0.05, **P < 0.01.

Our previous study analyzed whole cell lysates of MoDCs, indicating that targeting of DC-SIGN receptor on DCs by *P. gingivalis* minor fimbriated strains induced phosphorylation of Akt and FOXO1 (34). To assess the function of these signaling/transcription factors, we analyzed nuclear/cytoplasmic shuttling of Akt and FOXO1 by Western blotting. We show that infection of MoDCs with Mfa1-positive Pg381 and DPG3, but not Mfa1-negative MFI, significantly increased induction of p-Akt and p-FOXO1 in the cytoplasm and nucleus (Figure 1); however, the nuclear translocation of FOXO1 was significantly reduced in DCs infected with Pg381 and DPG3, in comparison to DCs infected with the Mfa1- negative MFI.

**Figure 2:**
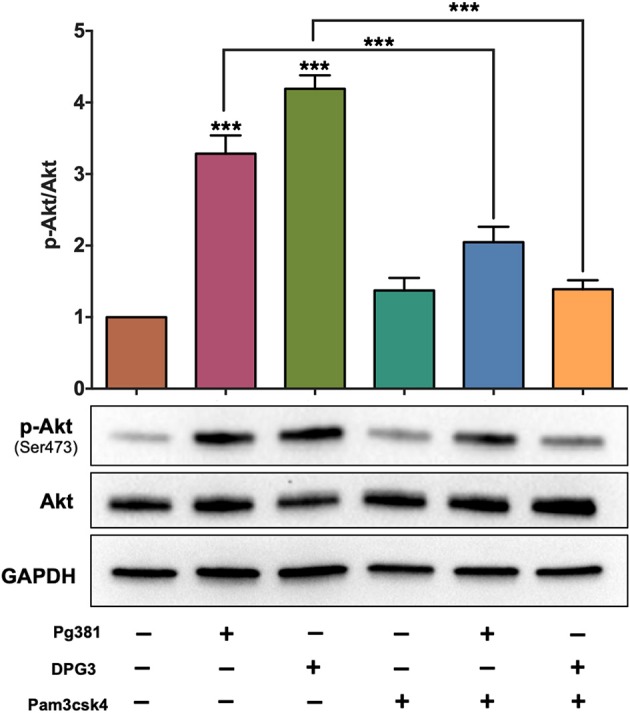
Selective activation of TLR1/2 attenuates the influence of DC-SIGN activation by *P. gingivalis* strains. Representative images of immunoblot and densitometric analysis of Akt and p-Akt. DCs were infected with *P. gingivalis* strains, Pg381, DPG, and MFI for 6 h. Some groups were treated with TLR1/2 ligand Pam3csk at the same time of infection. After normalization of the phosphorylated protein with total protein, the protein signal of control was set as 1, and the signals of other groups were normalized with control to calculate fold changes. GAPDH was used as internal control for normalizing the data. Data are expressed as mean ± SD. ***P < 0.001.

Targeting of DC-SIGN by the minor fimbria protein is known to affect FimA-TLR2 activation (22). Here we used the agonist Pam3csk4 (35) to determine a possible counter-regulatory role for TLR2 in overriding Mfa-1-DC-SIGN signaling. DCs were treated with 1 μg/mL Pam3csk4 at the same time of *P. gingivalis* infection. Pam3csk4 treatment significantly attenuated Akt phosphorylation/activation caused by *P. gingivalis* infection, nearly equivalent to uninfected control DCs (Figure 2).

### *P. gingivalis* Inhibits Autophagy in DCs by Targeting DC-SIGN Via an Akt/mTOR-Dependent Mechanism

**Figure 3:**
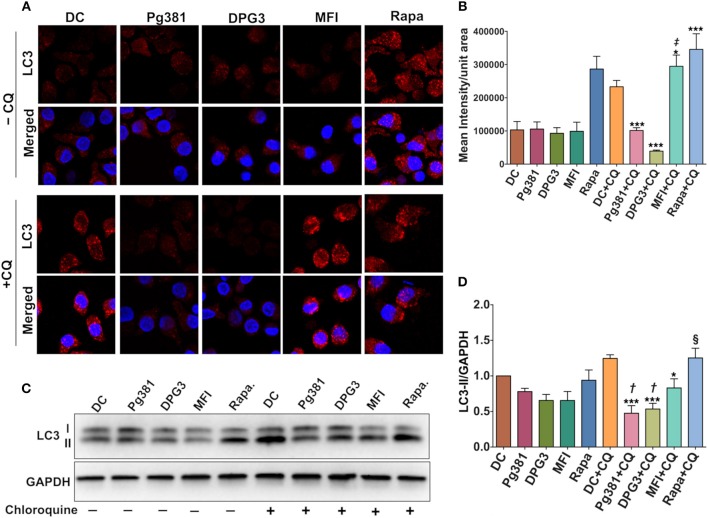
*P. gingivalis* minor fimbria decreases LC3B-II levels and autophagic flux. **(A)** Representative images of LC3 fluorescence staining. **(B)** Quantitative analysis of LC3 fluorescence intensity per unit area. **(C)** Representative images of immunoblot analysis of LC3B-II. **(D)** Densitometric analysis of LC3B-II. DCs were infected with *P. gingivalis* strains, Pg381, DPG, and MFI for 6 h. Some groups were treated with 1 μM rapamycin (Rapa). Each treatment was performed in duplicate to allow 5 μM chloroquine (CQ) to be added to each treatment group for the determination of LC3B-II steady state levels and autophagic flux. After normalization of LC3B-II to GAPDH, the protein signal of control was set as 1, and the signals of other groups were normalized and compared to control. Data are expressed as mean ± SD. *P < 0.05, ***P < 0.001 (DC+CQ vs. Pg381 + CQ, DPG3 + CQ, MFI + CQ, and Rapa). ‡*P* < 0.001 (Pg381 + CQ and DPG3 + CQ vs. MFI+CQ). †*P* < 0.01 (Pg381 + CQ and DPG3 + CQ vs. MFI + CQ). §*P* < 0.001 (Pg381 + CQ and DPG3 + CQ vs. Rapa + CQ).

We next sought to determine how Akt activation by *P. gingivalis* affects autophagy through immunofluorescence staining of punctate LC3B-II. As expected, LC3B is diffuse in cytosol when autophagy is inhibited or absent, but when autophagy is activated, lipidated LC3B, referred to as LC3B-II, localizes to the autophagosome membrane resulting in punctate cytoplasmic staining (36). As shown (Figures 3A,B), infection by Mfa1-positive Pg381 and DPG3 resulted in lower LC3 fluorescence (granular staining of LC3B) relative to uninfected DCs or DCs infected with Mfa1-negative strain MFI. A reduction in LC3B staining could indicate a reduction in autophagy or an enhancement of autophagic flux. We thus examined LC3B-II protein level after blocking autophagy flux using chloroquine (CQ) at a concentration of 5 uM. Blocking autolysosomal flux, allows steady state levels of LC3 to be compared between treatment groups. As shown in Figures 3C,D, LC3B-II significantly accumulated in uninfected DCs and in DCs treated with rapamycin after treatment with chloroquine. In contrast, LC3B-II levels did not accumulate to higher levels in DCs infected with Pg381 or DPG3, supporting role in autophagy suppression in these cells. MFI-infected cells showed a small increase in LCBII in the CQ-treated cells, establishing that these cells expressed some level of functional autophagy, but the level is significantly less than that in DC control cells. These studies revealed that DCs infected with *P. gingivalis* strains induced autophagy defects, in particular, involving downregulation of LC3B-II.

**Figure 4:**
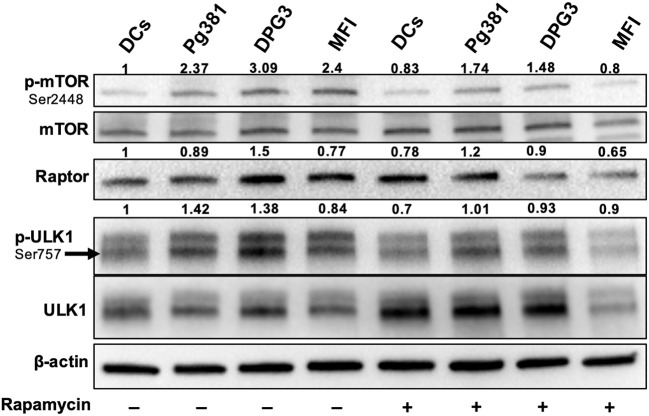
*P. gingivalis* inhibits autophagy in DCs via Akt/mTOR-dependent manner. Representative images of immunoblot analysis of mTOR, p-mTOR, Raptor, ULK-1, and p-ULK-1. DCs were infected with *P. gingivalis* strains Pg381, DPG, and MFI for 6 h. Some groups were treated with rapamycin 1 μM while infection with *P. gingivalis*. Total cells lysates were used for Western blotting analysis. After normalization of the phosphorylated protein with the corresponding total protein, the protein signal of control was set as 1, and the signals of other groups were normalized with control to calculate fold changes.

**Figure 5:**
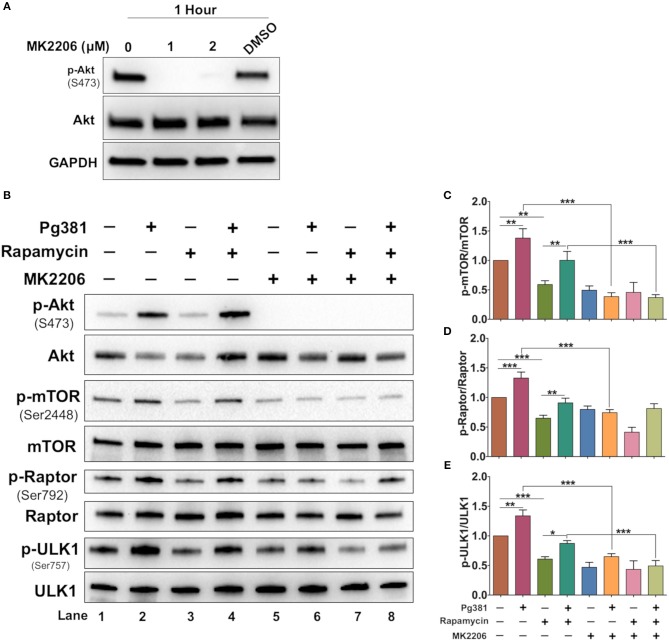
MK2206 abrogates the influence of *P. gingivalis* on DCs autophagy. **(A)** Representative images of immunoblot analysis of Akt and p-Akt. DCs were treated with Akt inhibitor, MK2206 for 1 h. **(B)** Representative images of immunoblot analysis of Akt and p-Akt, mTOR, p-mTOR, Raptor, p-Raptor, Ulk1, and p-ULK1. DCs were pretreated with Akt MK2206 for 1 h before infection with *P. gingivalis* Pg381 for 6 h. Total cells lysates were used for Western blotting analysis. **(C–E)** Densitometric analysis of p-mTOR, p-Raptor and p-ULK1, respectively. After normalization of the phosphorylated protein with the corresponding total protein, the protein signal of control was set as 1, and the signals of other groups were normalized with control to calculate fold changes. Data are expressed as mean ± SD. *P < 0.05, **P < 0.01, ***P < 0.001.

Akt/mTOR pathway, an important regulator of autophagy, was examined. Upon activation, Akt phosphorylates mTORC1, which in turn phosphorylates ULK1 at serine residue 757, which is known to inhibit autophagy (37). Infection with Mfa1-positive *P. gingivalis* strains induced expression of p-mTOR, p-ULK1 and Raptor (Figure 4), consistent with the conclusion that p-Akt activation blocks autophagy induction. Rapamycin, a small molecule inhibitor of mTOR (38) was used to induce autophagy as a positive control (Figure 4). Interestingly, *P. gingivalis*-infected MoDCs were resistant to rapamycin induced autophagy activation, relative to uninfected DCs or DCs infected with Mfa1-negative strain MFI (Figure 4). We postulated that activation of Akt under *P. gingivalis* infection was responsible (Figures 1, 2). To test this possibility, MK2206, an allosteric pan Akt inhibitor (39), was used. Initial dose response studies established level of inhibition of Akt phosphorylation by treating the cells for 1 h with 1 and 2 μM MK2206 (Figure 5). Subsequently, 1 μM MK2206 was used in all experiments in this study. As shown in previous figures, *P. gingivalis* infection alone induced phosphorylation of Akt, mTOR, Raptor and ULK1 (Figure 5, lane 2). While the expression level of all of these markers were significantly decreased with rapamycin treatment (Figure 5, lane 3), this effect was inhibited in DCs infected with *P. gingivalis* and treated with rapamycin (Figure 5, lane 4). In addition, MK2206 not only induced Akt inhibition, but also significantly decreased phosphorylation of mTOR, Raptor and ULK1 in uninfected (Figure 5, lane 5) and *P. gingivalis*-infected DCs (Figure 5, lane 6). These data provide strong support for the involvement of Akt/mTOR signaling in *P. gingivalis*-induced autophagy inhibition in DCs, involving DC-SIGN receptor.

### *P. gingivalis* Effectively Inhibits Apoptosis in DCs

**Figure 6:**
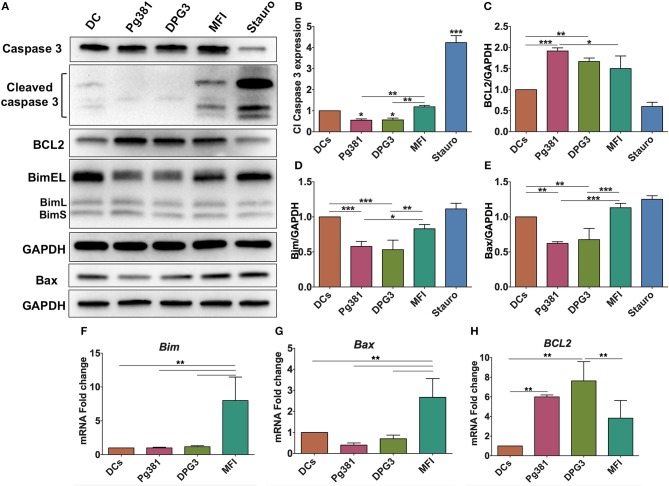
*P. gingivalis* inhibits apoptosis in DCs. **(A)** Representative images of immunoblot analysis of Cleaved caspase-3, Bcl2, Bim, and Bax. DCs were infected with *P. gingivalis* strains, Pg381, DPG, and MFI for 6 h and total cell lysates were subjected to Western blotting analysis. Staurosporine (Stauro) was used to as a positive control to induce apoptosis in DCs. **(B–E)** Densitometric analysis of Cleaved caspase-3, Bcl2, Bim, and Bax, respectively. After normalization with total GAPDH, the protein signal of control was set as 1, and the signals of other groups were normalized with control to calculate fold changes. Cleaved caspase-3 was normalized with total caspase-3. **(F–H)** mRNA expression of Bim, Bax, and Bcl2, respectively in uninfected DCs and DC infected with *P. gingivalis* strains. mRNA levels were measured by RT-PCR and normalized to GAPDH. Data are expressed as mean ± SD. *P < 0.05, **P < 0.01, ***P < 0.001.

Having established the consistent role of Akt activation by *P. gingivalis* infection, we next sought to identify common downstream effectors of p-Akt pro-survival action. Because Akt activation often impacts the level of caspase action in cells, the expression level of cleaved caspase-3 was evaluated. Caspase-3 plays an important role in the execution-phase of the apoptosis process and is activated by both caspase 8 and caspase 9 of the intrinsic and extrinsic apoptotic pathway (40). Western blotting identified a significant decrease in the expression level of cleaved caspase-3 when DCs were infected with Pg381 or DPG3, while infection with MFI increased cleaved caspase-3 expression (Figures 6A,B). Staurosporine, used as a positive control for induction of apoptosis showed the highest level of cleaved caspase-3. The expression level of Bcl2 and other members of the Bcl2 family that regulate apoptosis and autophagy in cells were also analyzed (41). DCs infected by Mfa-1 positive strains Pg381 and DPG3 increased the anti-apoptotic protein Bcl2 (Figures 6A,C) while decreasing the levels of the pro-apoptotic proteins Bim and Bax (Figures 6A,D,E). In contrast, DCs infected with Mfa-1 negative MFI infection did not show these alterations of the Bcl2 family members (Figures 6A,D,E). In parallel with protein analysis, qPCR data showed that the mRNA expression of the pro-apoptotic genes *Bax* and *Bim* were significantly upregulated when DCs were infected with MFI, but not with Pg381 or DPG3 (Figures 6F,G). On the other hand, *Bcl2* mRNA expression was significantly upregulated with Pg381 and DPG3, but not with MFI (Figure 6H).

**Figure 7:**
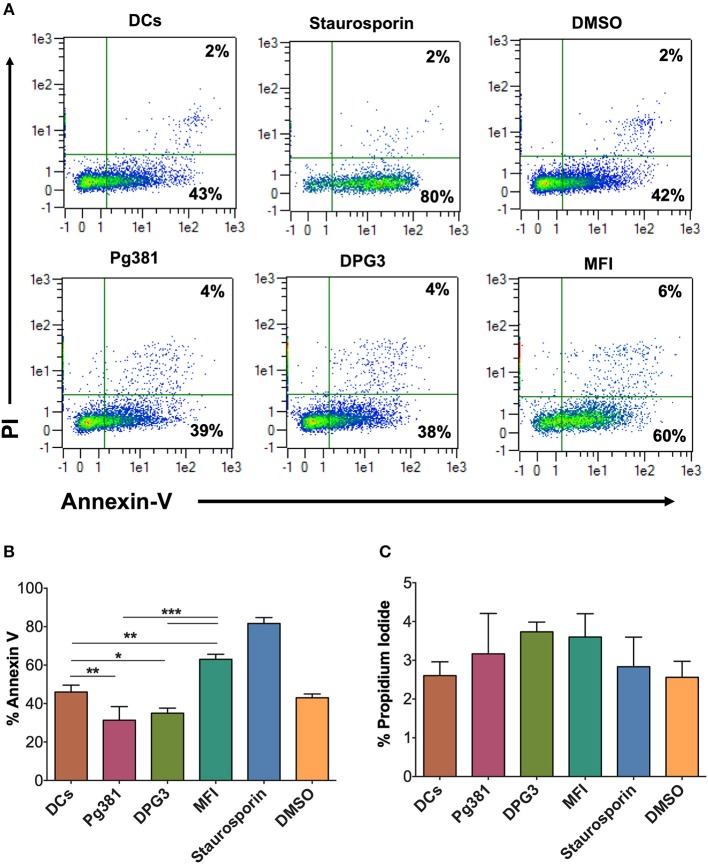
*P. gingivalis* infection induces an anti-apoptotic DC phenotype. **(A)** Representative flow cytometry dot plot figures of Annexin V vs. PI. DCs were infected with *P. gingivalis* strains, Pg381, DPG, and MFI for 12 h. Annexin V and PI staining was carried out according to the manufacturer recommendation. Staurosporine (Stauro) was used to as a positive control to induce apoptosis in DCs. **(B,C)** Percentage of expression of Annexin V and PI, respectively, in control DCs and DCs infected with *P. gingivalis* strains. Data are expressed as mean ± SD. *P < 0.05, **P < 0.01, ***P < 0.001.

To determine functionally the number of dead DCs among those infected by *P. gingivalis* strains, we analyzed the levels of propidium iodide (PI) uptake and Annexin V expression by flow cytometry. The results showed a decrease in % Annexin V in response to Mfa1 positive Pg381 and DPG3, but an increase in response to Mfa1-negative MFI (Figure 7). No significant differences were detected with PI staining, indicating that cells were not undergoing necrosis. Staurosporine dissolved in DMSO was used as a positive control for apoptosis in DCs. These combined studies provide strong evidence that infected cells are resistant to apoptosis, involving upregulation of Bcl2 as a means of survival.

### *P. gingivalis*-Induced Anti-apoptotic Effect in DCs Is Akt-Dependent and Is Attenuated by MK2206 and ABT-199

**Figure 8:**
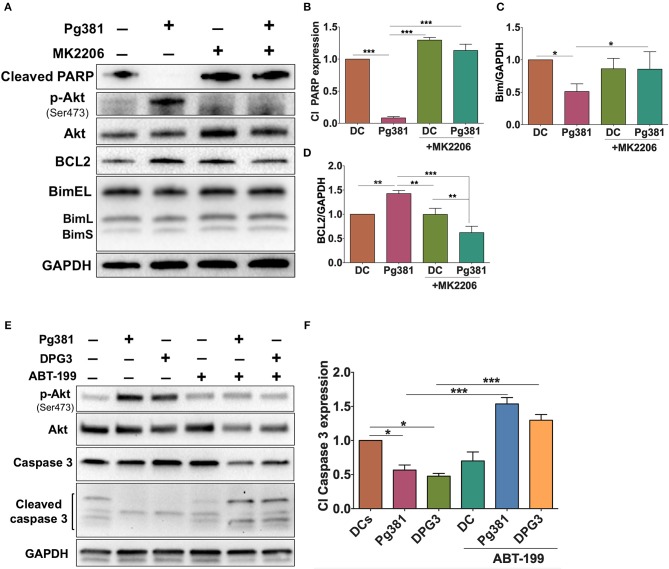
MK2206 and ABT-199 attenuate the influence of *P. gingivalis* on DCs apoptosis. **(A)** Representative images of immunoblot analysis of Cleaved-PARP, Akt, p-Akt, Bcl2, and Bim. **(B–D)** Densitometric analysis of Cleaved-PARP, Akt, p-Akt, Bcl2, and Bim, respectively. After normalization of Cleaved-PARP, Akt, p-Akt, Bcl2, and Bim with GAPDH, the protein signal of control was set as 1, and the signals of other groups were normalized with control to calculate fold changes. **(E)** Representative images of immunoblot analysis of Akt, p-Akt, caspase-3 and cleaved caspase-3. **(F)** Densitometric analysis of cleaved caspase-3. After normalization of p-Akt with total Akt and cleaved caspase-3 with caspase-3, the protein signal of control was set as 1, and the signals of other groups were normalized with control to calculate fold changes. DCs were infected with *P. gingivalis* strains, Pg381, or DPG3 for 6 h. Some groups were treated with MK2206 or ABT-199 at the same time of infection. Data are expressed as mean ± SD. *P < 0.05, **P < 0.01, ***P < 0.001.

To further elucidate the role of the Akt/Bcl2 axis in regulating the anti-apoptotic DC phenotype upon *P. gingivalis* infection, MK2206 was used to block Akt activation during *P. gingivalis* infection. *P. gingivalis* failed to induce DC survival upon Akt inhibition, evidenced by a significant increase in cleaved PARP and Bim, but also a decrease in Bcl2 expression (Figures 8A–D), further supporting a key role of Akt in the *P. gingival*-driven DC longevity.

If Bcl2 is protecting DCs from apoptosis, targeting Bcl2 should increase their susceptibility to *P. gingivalis*-induced apoptosis. ABT-199 (Venetoclax), a small molecule inhibitor that disrupts the interaction between heterodimer complex of antiapoptotic Bcl2 and its proapoptotic partners BAK/BAX, was used to attempt to specifically override the anti-apoptotic effect of Mfa1 in DCs as shown in Figures 8E,F. DCs were treated with 250 nM ABT-199 (42) concurrently with P*. gingivalis* infection. ABT-199 treatment resulted in a significant increase in cleaved caspase-3 in DCs infected with *P. gingivalis* strains, that previously showed resistance to apoptosis, relative to control DCs and DCs infected with *P. gingivalis* without ABT199 treatment (Figures 8E,F). Moreover, ABT-199 treatment also resulted in a marked decrease in Akt phosphorylation in *P. gingivalis*-infected DCs (Figures 8E,F).

## Discussion

Periodontal disease is a multifactorial disease, with unclear pathophysiologic mechanisms contributing to its development in susceptible hosts. Several pathogens are credited with the development of periodontal disease, however *P. gingivalis*, via its virulence factors, is considered a major etiological agent of the disease (43). The mechanism whereby the periodontal pathogen, *P. gingivalis* manipulates the host immune system is not totally understood. The present study aimed to elucidate the mechanism by which *P. gingivalis* affects the two important cell survival processes, apoptosis and autophagy, in DCs. Our results show that targeting the receptor DC-SIGN in DCs by *P. gingivalis*, via its minor fimbria, results in induction of pro-survival signaling pathways, namely the Akt/FOXO1 pathway. In addition, our results also revealed that *P. gingivalis* promotes differential Akt and FOXO1 cytoplasmic/nuclear shuttling pattern, depending on *P. gingivalis* fimbrial expression. When Akt is activated, it localizes to the nucleus, where it phosphorylates and inactivates FOXO1 (44). Upon phosphorylation, FOXO1 translocates to the cytoplasm, where it undergoes polyubiquitination and subsequently proteasomal degradation, leading to inhibition of apoptosis, as FOXO members transcriptionally regulate the activation of genes encoding pro-apoptotic proteins, such as Bim (45). Not only does FOXO1 regulate cell survival, but it also plays an important role in bacterial clearance and immune cell function. Interestingly, Dong et al., using *in vivo* and *in vitro* studies of FOXO1 effect on neutrophils in response to *P. gingivalis* infection, shows that *P. gingivalis* promotes nuclear localization of FOXO1 and this effect is dependent on TLR2 (46). This suggests the influence of *P. gingivalis* on FOXO1 in the absence of strong DC-SIGN signal, which is minimally expressed in neutrophils. On the other hand, our results showed that DC-SIGN activation promotes phosphorylation of Akt and FOXO1, suggesting an override of the TLR2 signal by DC-SIGN in DCs under the influence of *P. gingivalis* minor fimbria as a strategy to evade innate immune response. This hypothesis was confirmed by activating TLR2 via Pam3csk4 concurrent with *P. gingivalis* infection. TLR2 overactivation by this route led to a significant decrease in the expression level of p-Akt. Dampening TLR2 signaling by *P. gingivalis* could be a tactic to increase the survival of its host cell, and disarm the immune response, as TLR2 signal is required for production of proinflammatory cytokines (47).

Autophagy is a process whereby the cell degrades its intracellular damaged proteins and organelles by sequestering and directing cargo to the lysosome. Autophagy is pivotal for the cell to maintain cellular homeostasis and defense against invading pathogens. By targeting intracellular bacteria to lysosomes, autophagy comprises an important element of innate immune response which is the first line of defense. Our study showed that steady state LC3B-II levels decrease upon infecting DCs with minor fimbriated *P. gingivalis* strains, suggesting inhibition of the autophagy pathway. Autophagy plays a crucial role in antigen processing leading to antigen presentation (48, 49), moreover previous reports of decrease in MHC class II expression on DCs from periodontitis patients (50) and in DCs infected with *P. gingivalis* (30), emphasizes the relevance of our findings to antigen presentation *in vivo*. Interestingly, several pathogens have evolved multiple ways of exploiting the host-autophagic machinery for their benefit. While *Salmonella, Mycobacterium, Brucella* and *Legionella* use the host autophagic vacuoles for replication and persistence, *Shigella* and *Listeria* escape the host autophagy process and live in the nutritionally rich cytosol (51–53). The differential utilization of the host autophagy by *P. gingivalis* has been studied before. Recently, it has been reported that *P. gingivalis* LPS, which is a ligand for TLR2, promotes autophagy of human gingival fibroblasts (54). Although previous studies demonstrated that *P. gingivalis* induces autophagy in human gingival epithelial cells for its survival (55), our previous studies have shown that it manipulates the autophagy process to survive within DCs and allow escape from immune surveillance (29). The influence of autophagy on *P. gingivalis* survival within the host is apparently a host-cell type- and bacteria strain-dependent phenomenon (56–58).

The Akt/mTOR signaling axis is an important regulator of autophagy and crucial for many aspects of cell survival during physiologic and pathologic conditions. Our results show that targeting DC-SIGN on DCs by *P. gingivalis* minor fimbria induces activation of the Akt/mTOR/ULK1 pathway. Recently, Zhao et al. demonstrated that autophagy via activation of mTOR/ULK1 enhances the killing of invading *Salmonella* (59). Thus, our study revealed that DCs infected with minor fimbriated *P. gingivalis* strains showed resistance to rapamycin-induced autophagy. The mechanism of *P. gingivalis*-infected DCs resistance to rapamycin is still unclear, however it could be attributed to the constitutive activation of Akt (Figure 2). Furthermore, inhibition of Akt by MK2206 suggest that *P. gingivalis* subverts autophagy in DCs via Akt/mTOR-dependent manner. We showed that inhibition of Akt abrogates the positive influence of *P. gingivalis* on mTOR activation, decreasing phosphorylation of mTOR and its downstream effector ULK1, leading to activation of ULK1 and the autophagy process.

The results of the present study suggest that *P. gingivalis* minor fimbria induces dysregulation of autophagy and apoptosis in DCs. Apoptosis is a process by which unwanted cells are eliminated, crucial to mounting a homeostatic immune response. In this study, the regulatory role of *P. gingivalis* minor fimbria on DCs apoptosis was examined by multiple approaches, including Western blot analysis of multiple proteins involved in cell survival and death, ICC, and evaluation of phosphatidylserine localization by Annexin V labeling. In live cells, phosphatidylserine is localized to the inner leaflet of the plasma membrane. When cells engage in early stages of apoptosis, phosphatidylserine localizes to the outer leaflet due to loss of membrane asymmetry. Annexin V binds to the exposed phosphatidylserine in a calcium-dependent manner. We showed that targeting DC-SIGN leads to a decrease in Annexin V expression, suggesting inhibition of apoptosis. Perturbation of DC apoptosis can result in dire consequences such as development of autoimmune diseases (60). The Bcl2 family of proteins governs the intrinsic pathway of apoptosis. Apoptosis is inhibited when the proapoptotic members of Bcl2 family insert their BH3 domain into the hydrophobic groove, former by BH1-4 domains, of the antiapoptotic member Bcl2 (61). This interaction prevents dimerization of the antiapoptotic partners at the outer mitochondrial membrane, leading to inhibition of Cytochrome C release from the mitochondria and cleavage of executioner caspases, including caspase-3, hence apoptosis process is inhibited.

We further showed that *P. gingivalis* minor fimbriated strains induce an increase in the anti-apoptotic Bcl2 protein expression and a decrease in pro-apoptotic proteins Bim, Bax and cleaved caspase-3, promoting survival of DCs. Importantly, deficiency in Bim has been contributed to breakdown of immune tolerance and the development of autoimmune diseases in Bim knockout mice (62). In addition, the increase in Bcl2 expression shown in Figure 6 could be attributed to the increase in Akt activation, as suggested previously by Pugazhenthi et al. (63).

Consistent with a key role for Bcl2 overexpression in DC survival was the ability of ABT-199 to induce apoptosis in *P. gingivalis-*infected DCs. ABT-199 is a small molecule inhibitor that selectively binds to Bcl2 and currently is being used in clinical trials for cancer treatment (42). Our results also showed that ABT-199 treatment decreased phosphorylation of Akt in *P. gingivalis*-infected DCs, consistent with findings of previous work that suggested regulation of cell survival via Bcl2-mediated activation of Akt (64). Using *in vitro* and *in vivo* experiments, Zhan et al. described the potency of ABT199 to kill plasmacytoid DCs associated with systemic lupus erythematosus (65). These findings together suggest the possibility of using pharmacological agents as a new modality to inhibit prolonged survival of *P. gingivalis*-infected DCs.

Abnormal survival of microbe-loaded DCs is expected to contribute to systemic inflammation and microbial dissemination to distant sites. Carrion et al., using post-mortem analysis of coronary artery samples from patients with coronary artery disease and periodontal disease, showed localization of *P. gingivalis*-infected DCs in atherosclerotic plaques and co-localization of DCs marker, DC-SIGN, with *P. gingivalis* minor fimbria protein Mfa-1 (12). This could be a result of *P. gingivalis* exploiting migrating DCs, endowed with impaired pathogen clearance and extended survival, to travel to permissive distant sites.

**Figure 9:**
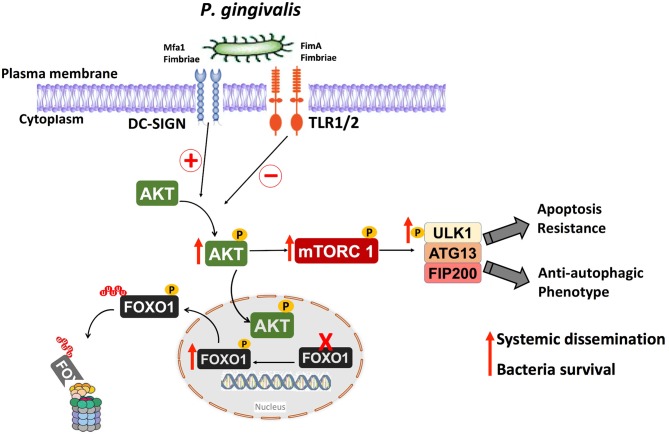
Anti-autophagic and anti-apoptotic pathway activated in human DCs by *P. gingivalis* and its fimbria proteins. Targeting the receptor DC-SIGN on human DCs by *P. gingivalis* Mfa1 fimbria results in the activation of Akt, which phosphorylates and inactivates FOXO1 resulting in anti-apoptotic DCs phenotype, thereby extending the lifespan of host DCs. The activation of Akt results also in activation of Akt/mTOR signaling axis, which inhibits antimicrobial autophagic machinery in human DCs, resulting in survival of intracellular *P. gingivalis*.

In summary, considering the limitations of the present *in vitro* study, we have identified important roles for the fimbrial phenotype of *P. gingivalis* in activation of a signaling pathway involved in downregulation of both apoptosis and autophagy in DCs. This pathways is shown diagrammatically in Figure 9. It is noteworthy that the absence of a functional DC-SIGN ortholog in murine DCs (66–68) makes it difficult to confirm these results in mouse model of infection; however, this same pathway has been shown to be activated *in vivo* in gingival tissues and blood cells of subjects with periodontitis (34). In addition, our recent clinical pilot study has shown that periodontitis (PD) patients exhibit low autophagic profile and decreased autophagy-related proteins expression relative to healthy subjects (69). The same study also shows that placing periodontitis patients on Vitamin D supplementation, which supports an anti-inflammatory, pro-autophagy environment restores autophagy and increases the expression of the autophagy-related proteins in PD patients. We would thus suggest that efforts to restore immune homeostasis in PD patients consider the promotion of antimicrobial autophagy and apoptosis to prevent untoward inflammatory responses in the periphery and increase bacterial clearance.

## Data Availability Statement

The raw data supporting the conclusions of this manuscript will be made available by the authors, without undue reservation, to any qualified researcher.

## Ethics Statement

*In vitro* monocyte-derived DCs (MoDCs) studies were determined by the Human Assurance Committee at Augusta University to be human subject exempt, due to the use of anonymized peripheral blood samples for monocytes.

## Author Contributions

MM, PS, and CC conceived and designed the research. MM, OT, MR, and ME performed experiments. MM, PS, and CC analyzed the data and interpreted the results. MM and CC prepared figures and drafted the manuscript. MM, RA, DF, PS, and CC edited and revised the manuscript. All authors approved the final manuscript version.

### Conflict of Interest

The authors declare that the research was conducted in the absence of any commercial or financial relationships that could be construed as a potential conflict of interest.

## References

[1] Eke PI Dye BA Wei L Slade GD Thornton-Evans GO Borgnakke WS. Update on prevalence of periodontitis in adults in the United States: NHANES 2009 to 2012. J Periodontol. (2015) 86:611–22. 10.1902/jop.2015.140520 25688694 PMC4460825

[2] Herrero ER Fernandes S Verspecht T Ugarte-Berzal E Boon N Proost P. Dysbiotic biofilms deregulate the periodontal inflammatory response. J Dent Res. (2018) 97:547–55. 10.1177/0022034517752675 29394879

[3] Lamont RJ Hajishengallis G. Polymicrobial synergy and dysbiosis in inflammatory disease. Trends Mol Med. (2015) 21:172–83. 10.1016/j.molmed.2014.11.004 25498392 PMC4352384

[4] Ebersole JL Dawson D III Emecen-Huja P Nagarajan R Howard K Grady ME. The periodontal war: microbes and immunity. Periodontology. 2000. (2017) 75:52–115. 10.1111/prd.12222 28758303

[5] Hajishengallis G Korostoff JM. Revisiting the Page & Schroeder model: the good, the bad and the unknowns in the periodontal host response 40 years later. Periodontology. (2017) 75:116–51. 10.1111/prd.12181 28758305 PMC5539911

[6] Cutler CW Jotwani R. Dendritic cells at the oral mucosal interface. J Dent Res. (2006) 85:678–89. 10.1177/154405910608500801 16861283 PMC2254185

[7] Niess JH Reinecker HC. Dendritic cells: the commanders-in-chief of mucosal immune defenses. Curr Opin Gastroenterol. (2006) 22:354–60. 10.1097/01.mog.0000231807.03149.54 16760749

[8] Hajishengallis G Darveau RP Curtis MA. The keystone-pathogen hypothesis. Nat Rev Microbiol. (2012) 10:717–25. 10.1038/nrmicro2873 22941505 PMC3498498

[9] Jotwani R Cutler CW. Multiple dendritic cell (DC) subpopulations in human gingiva and association of mature DCs with CD4^+^ T-cells *in situ*. J Dent Res. (2003) 82:736–41. 10.1177/154405910308200915 12939360

[10] Jotwani R Muthukuru M Cutler CW. Increase in HIV receptors/co-receptors/alpha-defensins in inflamed human gingiva. J Dent Res. (2004) 83:371–7. 10.1177/154405910408300504 15111627

[11] Jotwani R Palucka AK Al-Quotub M Nouri-Shirazi M Kim J Bell D. Mature dendritic cells infiltrate the T cell-rich region of oral mucosa in chronic periodontitis: *in situ, in vivo*, and *in vitro* studies. J Immunol. (2001) 167:4693–700. 10.4049/jimmunol.167.8.4693 11591800 PMC3739284

[12] Carrion J Scisci E Miles B Sabino GJ Zeituni AE Gu Y. Microbial carriage state of peripheral blood dendritic cells (DCs) in chronic periodontitis influences DC differentiation, atherogenic potential. J. Immunol. (2012) 189:3178–87. 10.4049/jimmunol.1201053 22891282 PMC3459682

[13] Velsko IM Chukkapalli SS Rivera MF Lee JY Chen H Zheng D. Active invasion of oral and aortic tissues by *Porphyromonas gingivalis* in mice causally links periodontitis and atherosclerosis. PLoS ONE. (2014) 9:e97811. 10.1371/journal.pone.0097811 24836175 PMC4024021

[14] Hajishengallis G Wang M Bagby GJ Nelson S. Importance of TLR2 in early innate immune response to acute pulmonary infection with *Porphyromonas gingivalis* in mice. J. Immunol. (2008) 181:4141–9. 10.4049/jimmunol.181.6.4141 18768871 PMC2625304

[15] Poole S Singhrao SK Kesavalu L Curtis MA Crean S. Determining the presence of periodontopathic virulence factors in short-term postmortem Alzheimer's disease brain tissue. J Alzheimers Dis. (2013) 36:665–77. 10.3233/JAD-121918 23666172

[16] Sugiyama S Takahashi SS Tokutomi FA Yoshida A Kobayashi K Yoshino F Gingival vascular functions are altered in type 2 diabetes mellitus model and/or periodontitis model. J Clin Biochem Nutr. (2012) 51:108–13. 10.3164/jcbn.11-103 22962527 PMC3432819

[17] Liang S Ren H Guo H Xing W Liu C Ji Y. Periodontal infection with *Porphyromonas gingivalis* induces preterm birth and lower birth weight in rats. Mol Oral Microbiol. (2018) 33:312–21. 10.1111/omi.12227 29754448

[18] Dasanayake AP Boyd D Madianos PN Offenbacher S Hills E. The association between *Porphyromonas gingivalis*-specific maternal serum IgG and low birth weight. J Periodontol. (2001) 72:1491–7. 10.1902/jop.2001.72.11.1491 11759860

[19] Enersen M Nakano K Amano A. *Porphyromonas gingivalis* fimbriae. J Oral Microbiol. (2013) 5:20265. 10.3402/jom.v5i0.20265 23667717 PMC3647041

[20] Zeituni AE Jotwani R Carrion J Cutler CW. Targeting of DC-SIGN on human dendritic cells by minor fimbriated *Porphyromonas gingivalis* strains elicits a distinct effector T cell response. J Immunol. (2009) 183:5694–704. 10.4049/jimmunol.0901030 19828628 PMC2770168

[21] Zeituni AE McCaig W Scisci E Thanassi DG Cutler CW. The native 67-kilodalton minor fimbria of *Porphyromonas gingivalis* is a novel glycoprotein with DC-SIGN-targeting motifs. J Bacteriol. (2010) 192:4103–10. 10.1128/JB.00275-10 20562309 PMC2916435

[22] Davey M Liu X Ukai T Jain V Gudino C Gibson FC III. Bacterial fimbriae stimulate proinflammatory activation in the endothelium through distinct TLRs. J Immunol. (2008) 180:2187–95. 10.4049/jimmunol.180.4.2187 18250425

[23] Hajishengallis G Wang M Liang S Triantafilou M Triantafilou K. Pathogen induction of CXCR4/TLR2 cross-talk impairs host defense function. Proc Natl Acad Sci USA. (2008) 105:13532–7. 10.1073/pnas.0803852105 18765807 PMC2533224

[24] Oh JE Lee HK. Pattern recognition receptors and autophagy. Front Immunol. (2014) 5:300. 10.3389/fimmu.2014.00300 25009542 PMC4070062

[25] Uhl M Kepp O Jusforgues-Saklani H Vicencio JM Kroemer G Albert ML. Autophagy within the antigen donor cell facilitates efficient antigen cross-priming of virus-specific CD8^+^ T cells. Cell Death Different. (2009) 16:991–1005. 10.1038/cdd.2009.8 19229247

[26] Fiegl D Kagebein D Liebler-Tenorio EM Weisser T Sens M Gutjahr M. Amphisomal route of MHC class I cross-presentation in bacteria-infected dendritic cells. J Immunol. (2013) 190:2791–806. 10.4049/jimmunol.1202741 23418629

[27] Wildenberg ME Vos AC Wolfkamp SC Duijvestein M Verhaar AP Te Velde AA. Autophagy attenuates the adaptive immune response by destabilizing the immunologic synapse. Gastroenterology. (2012) 142:1493–503.e6. 10.1053/j.gastro.2012.02.034 22370477

[28] Lamhamedi-Cherradi SE Zheng SJ Maguschak KA Peschon J Chen YH. Defective thymocyte apoptosis and accelerated autoimmune diseases in TRAIL-/- mice. Nat. Immunol. (2003) 4:255–60. 10.1038/ni894 12577054

[29] El-Awady AR Miles B Scisci E Kurago ZB Palani CD Arce RM. *Porphyromonas gingivalis* evasion of autophagy and intracellular killing by human myeloid dendritic cells involves DC-SIGN-TLR2 crosstalk. PLoS Pathog. (2015) 10:e1004647. 10.1371/journal.ppat.1004647 25679217 PMC4352937

[30] Miles B Scisci E Carrion J Sabino GJ Genco CA Cutler CW. Noncanonical dendritic cell differentiation and survival driven by a bacteremic pathogen. J Leukoc Biol. (2013) 94:281–9. 10.1189/jlb.0213108 23729500 PMC3714568

[31] Arjunan P El-Awady A Dannebaum RO Kunde-Ramamoorthy G Cutler CW. High-throughput sequencing reveals key genes and immune homeostatic pathways activated in myeloid dendritic cells by *Porphyromonas gingivalis* 381 and its fimbrial mutants. Mol Microbiol. (2016) 31:78–93. 10.1111/omi.12131 26466817 PMC5486950

[32] Takahashi Y Davey M Yumoto H Gibson FC III Genco CA. Fimbria-dependent activation of pro-inflammatory molecules in *Porphyromonas gingivalis* infected human aortic endothelial cells. Cell Microbiol. (2006) 8:738–57. 10.1111/j.1462-5822.2005.00661.x 16611224

[33] Cutler CW Kalmar JR Arnold RR Phagocytosis of virulent *Porphyromonas gingivalis* by human polymorphonuclear leukocytes requires specific immunoglobulin G. Infect Immun. (1991) 59:2097–104. 2037370 10.1128/iai.59.6.2097-2104.1991 PMC257971

[34] Arjunan P Meghil MM Pi W Xu J Lang L El-Awady A. Oral pathobiont activates anti-apoptotic pathway, promoting both immune suppression and oncogenic cell proliferation. Sci Rep. (2018) 8:16607. 10.1038/s41598-018-35126-8 30413788 PMC6226501

[35] Hara H Seregin SS Yang D Fukase K Chamaillard M Alnemri ES. The NLRP6 inflammasome recognizes lipoteichoic acid and regulates gram-positive pathogen infection. Cell. (2018) 175:1651–64.e14. 10.1016/j.cell.2018.09.047 30392956 PMC6294477

[36] Rosenfeldt MT Nixon C Liu E Mah LY Ryan KM. Analysis of macroautophagy by immunohistochemistry. Autophagy. (2012) 8:963–9. 10.4161/auto.20186 22562096 PMC3427261

[37] Kim J Kundu M Viollet B Guan KL. AMPK and mTOR regulate autophagy through direct phosphorylation of Ulk1. Nat Cell Biol. (2011) 13:132–41. 10.1038/ncb2152 21258367 PMC3987946

[38] Brown EJ Albers MW Shin TB Ichikawa K Keith CT Lane WS. A mammalian protein targeted by G1-arresting rapamycin-receptor complex. Nature. (1994) 369:756–8. 10.1038/369756a0 8008069

[39] Hirai H Sootome H Nakatsuru Y Miyama K Taguchi S Tsujioka K. MK-2206, an allosteric Akt inhibitor, enhances antitumor efficacy by standard chemotherapeutic agents or molecular targeted drugs *in vitro* and *in vivo*. Mol Cancer Ther. (2010) 9:1956–67. 10.1158/1535-7163.MCT-09-1012 20571069

[40] Schlegel J Peters I Orrenius S Miller DK Thornberry NA Yamin TT. CPP32/apopain is a key interleukin 1 beta converting enzyme-like protease involved in Fas-mediated apoptosis. J Biol Chem. (1996) 271:1841–4. 10.1074/jbc.271.4.1841 8567626

[41] Murphy KM Ranganathan V Farnsworth ML Kavallaris M Lock RB. Bcl-2 inhibits Bax translocation from cytosol to mitochondria during drug-induced apoptosis of human tumor cells. Cell Death Different. (2000) 7:102–11. 10.1038/sj.cdd.4400597 10713725

[42] Souers AJ Leverson JD Boghaert ER Ackler SL Catron ND Chen J. ABT-199, a potent and selective BCL-2 inhibitor, achieves antitumor activity while sparing platelets. Nat Med. (2013) 19:202–8. 10.1038/nm.3048 23291630

[43] Socransky SS Haffajee AD. Periodontal microbial ecology. Periodontology. (2005) 38:135–87. 10.1111/j.1600-0757.2005.00107.x 15853940

[44] Brunet A Bonni A Zigmond MJ Lin MZ Juo P Hu LS. Akt promotes cell survival by phosphorylating and inhibiting a Forkhead transcription factor. Cell. (1999) 96:857–68. 10.1016/S0092-8674(00)80595-4 10102273

[45] Birkenkamp KU Coffer PJ. FOXO transcription factors as regulators of immune homeostasis: molecules to die for? J Immunol. (2003) 171:1623–9. 10.4049/jimmunol.171.4.1623 12902457

[46] Dong G Song L Tian C Wang Y Miao F Zheng J. FOXO1 regulates bacteria-induced neutrophil activity. Front Immunol. (2017) 8:1088. 10.3389/fimmu.2017.01088 28928749 PMC5591501

[47] Huang LY Ishii KJ Akira S Aliberti J Golding B. Th1-like cytokine induction by heat-killed Brucella abortus is dependent on triggering of TLR9. J Immunol. (2005) 175:3964–70. 10.4049/jimmunol.175.6.3964 16148144

[48] Zhou D Li P Lin Y Lott JM Hislop AD Canaday DH. Lamp-2a facilitates MHC class II presentation of cytoplasmic antigens. Immunity. (2005) 22:571–81. 10.1016/j.immuni.2005.03.009 15894275

[49] Schmid D Pypaert M Munz C. Antigen-loading compartments for major histocompatibility complex class II molecules continuously receive input from autophagosomes. Immunity. (2007) 26:79–92. 10.1016/j.immuni.2006.10.018 17182262 PMC1805710

[50] Cury PR Carmo JP Horewicz VV Santos JN Barbuto JA. Altered phenotype and function of dendritic cells in individuals with chronic periodontitis. Arch Oral Biol. (2013) 58:1208–16. 10.1016/j.archoralbio.2013.03.013 23623310

[51] Celli J. The changing nature of the Brucella-containing vacuole. Cell Microbiol. (2015) 17:951–8. 10.1111/cmi.12452 25916795 PMC4478208

[52] Brumell JH Brucella hitches a ride with autophagy. Cell Host Microbe. (2012) 11:2–4. 10.1016/j.chom.2012.01.003 22264507

[53] Jo EK Yuk JM Shin DM Sasakawa C. Roles of autophagy in elimination of intracellular bacterial pathogens. Front Immunol. (2013) 4:97. 10.3389/fimmu.2013.00097 23653625 PMC3644824

[54] Liu J Wang X Zheng M Luan Q. Lipopolysaccharide from *Porphyromonas gingivalis* promotes autophagy of human gingival fibroblasts through the PI3K/Akt/mTOR signaling pathway. Life Sci. (2018) 211:133–9. 10.1016/j.lfs.2018.09.023 30218719

[55] Lee K Roberts JS Choi CH Atanasova KR Yilmaz O. *Porphyromonas gingivalis* traffics into endoplasmic reticulum-rich-autophagosomes for successful survival in human gingival epithelial cells. Virulence. (2018) 9:845–59. 10.1080/21505594.2018.1454171 29616874 PMC5955440

[56] Lamont RJ Chan A Belton CM Izutsu KT Vasel D Weinberg A. *Porphyromonas gingivalis* invasion of gingival epithelial cells. Infect Immun. (1995) 63:3878–85. 7558295 10.1128/iai.63.10.3878-3885.1995 PMC173546

[57] Dorn BR Dunn WA Jr Progulske-Fox A. Bacterial interactions with the autophagic pathway. Cell Microbiol. (2002) 4:1–10. 10.1046/j.1462-5822.2002.00164.x 11856168

[58] Rodrigues PH Reyes L Chadda AS Belanger M Wallet SM Akin D. *Porphyromonas gingivalis* strain specific interactions with human coronary artery endothelial cells: a comparative study. PLoS ONE. (2012) 7:e52606. 10.1371/journal.pone.0052606 23300720 PMC3530483

[59] Zhao X Tang X Guo N An Y Chen X Shi C. Biochanin a enhances the defense against *Salmonella enterica* infection through AMPK/ULK1/mTOR-mediated autophagy and extracellular traps and reversing SPI-1-Dependent Macrophage (MPhi) M2 polarization. Front Cell Infect Microbiol. (2018) 8:318. 10.3389/fcimb.2018.00318 30271755 PMC6142880

[60] Chen M Wang YH Wang Y Huang L Sandoval H Liu YJ. Dendritic cell apoptosis in the maintenance of immune tolerance. Science. (2006) 311:1160–4. 10.1126/science.1122545 16497935

[61] Kale J Osterlund EJ Andrews DW. BCL-2 family proteins: changing partners in the dance towards death. Cell Death Different. (2018) 25:65–80. 10.1038/cdd.2017.186 29149100 PMC5729540

[62] Chen M Huang L Wang J. Deficiency of Bim in dendritic cells contributes to overactivation of lymphocytes and autoimmunity. Blood. (2007) 109:4360–7. 10.1182/blood-2006-11-056424 17227827 PMC1885484

[63] Pugazhenthi S Nesterova A Sable C Heidenreich KA Boxer LM Heasley LE. Akt/protein kinase B up-regulates Bcl-2 expression through cAMP-response element-binding protein. J Biol Chem. (2000) 275:10761–6. 10.1074/jbc.275.15.10761 10753867

[64] Mortenson MM Galante JG Gilad O Schlieman MG Virudachalam S Kung HJ. BCL-2 functions as an activator of the AKT signaling pathway in pancreatic cancer. J Cell Biochem. (2007) 102:1171–9. 10.1002/jcb.21343 17960583

[65] Zhan Y Carrington EM Ko HJ Vikstrom IB Oon S Zhang JG. Bcl-2 antagonists kill plasmacytoid dendritic cells from lupus-prone mice and dampen interferon-alpha production. Arthritis Rheumatol. (2015) 67:797–808. 10.1002/art.38966 25418983

[66] Powlesland AS Ward EM Sadhu SK Guo Y Taylor ME Drickamer K. Widely divergent biochemical properties of the complete set of mouse DC-SIGN-related proteins. J Biol Chem. (2006) 281:20440–9. 10.1074/jbc.M601925200 16682406

[67] Caminschi I Corbett AJ Zahra C Lahoud M Lucas KM Sofi M. Functional comparison of mouse CIRE/mouse DC-SIGN and human DC-SIGN. Int Immunol. (2006) 18:741–53. 10.1093/intimm/dxl011 16569675 PMC7185610

[68] Garcia-Vallejo JJ van Kooyk Y. The physiological role of DC-SIGN: a tale of mice and men. Trends Immunol. (2013) 34:482–6. 10.1016/j.it.2013.03.001 23608151

[69] Meghil MM Hutchens L Raed A Multani NA Rajendran M Zhu H. The influence of vitamin D supplementation on local and systemic inflammatory markers in periodontitis patients: a pilot study. Oral Dis. (2019) 25:1403–13. 10.1111/odi.13097 30912231 PMC8796207

